# Effects of targets embedded within words in a visual search task

**DOI:** 10.2478/v10053-008-0150-9

**Published:** 2014-02-20

**Authors:** Jeremy W. Grabbe

**Affiliations:** Psychology Department, State University of New York, Plattsburgh, USA

**Keywords:** distracters, visual search, holistic bias, word frequency effects

## Abstract

Visual search performance can be negatively affected when both targets and distracters share a dimension relevant to the task. This study examined if visual search performance would be influenced by distracters that affect a dimension irrelevant from the task. In Experiment 1 within the letter string of a letter search task, target letters were embedded within a word. Experiment 2 compared targets embedded in words to targets embedded in nonwords. Experiment 3 compared targets embedded in words to a condition in which a word was present in a letter string, but the target letter, although in the letter string, was not embedded within the word. The results showed that visual search performance was negatively affected when a target appeared within a high frequency word. These results suggest that the interaction and effectiveness of distracters is not merely dependent upon common features of the target and distracters, but can be affected by word frequency (a dimension not related to the task demands).

## Introduction

The use of visual search tasks allows the study of both the characteristics (features) of a distracter and the relationship between the distracter and the target.

In the area of research on distraction, one question that is asked is “what qualities or properties of a distracter are processed?” Considerable evidence suggests that distracters can interfere by having common qualities with the target, such as color (Stroop, 1935) or orientation (Joseph & Optican, 1996). Others have looked at adjacent distracters (proximity) and their influence (Flowers & Wilcox, 1982).

What causes distracters to be detrimental to task performance? From a biological perspective, a visual search task detriment may stem from the utilization of a single brain area such as a common area of activity for spatial attention and spatial working memory (Silk, Bellgrove, Wrafter, Mattingly, & Cunnington, 2010). From a behavioral perspective, if dimensions of distracters overlap with a target, performance will be impaired. This impairment would stem from a competition for attention resources. In addition to the relevant distracters, Theeuwes (1991), for example, found that attention can be captured by singleton, irrelevant distracters that stand out by one feature among more homogeneous non-singletons but on a task-irrelevant dimension, such as color, in a parallel visual search task of targets based on shape. Does a distracter’s influence vary if its dissimilarity from the target is not about distinctiveness of features or relation to the target such as in Theeuwes’s study, but reflects a form of processing of information different from task demands? One of the questions asked in this study is what if the distracters were similar to the target (both target and distracters were letters), but formed a word? Would word recognition impede letter-level processing (task-relevant processing) due to holistic bias of word form processing (task-irrelevant processing)?

How would holistic word bias lead to impairment in target letter identification? Word recognition itself is automatic (Brown, Gore, & Carr, 2002). In the realm of word recognition, numerous studies have shown that words that occur in higher frequencies are more likely to be processed holistically (Allen, Wallace, & Weber, 1995). Holistic processing supersedes letter-level processing because of its faster and parsimonious nature.

In a task requiring more effortful attention, such as a visual search task, distracters that are processed automatically may provide more interference. In a visual search task in which a subject is searching for an A that is present in a letter string with a high-frequency word, such as *BXCANUT*, the reaction time may be slower than if searching for O in a string with a low-frequency word, like *BXCODUT*. A performance detriment for higher frequency words would provide evidence that automatically processed distracters can be more detrimental in a task, such as visual search, even when the task demands are not lexical, but based on selectivity of attention to features.

Bypassing letter-level processing (which is not predisposed to automatic semantic activation, see Friedrich, Henik, & Tzelgov, 1991) in preference to holistic word-form processing may cause a detriment in performance because recognition of a target letter would be obscured by word-form. This would be more of a case of not seeing the tree because of the forest as opposed to the customary “can’t see the forest for the trees.” In line with this possibility, interference in visual search can be created by globally defined distracters (Rauschenberger & Yantis, 2001; Suzuki & Cavanagh, 1995). Does automatic word recognition carry with it the letter-level data necessary for effective letter search performance? Or does the automatic word recognition impair performance by preventing or circumventing letter level processing?

Visual search tasks require more effort for attention control when distracters are randomized and unpredictable (Michael, Kiefer, & Niedeggen, 2012; Neo & Chua, 2006). Would the increase for attention required in a search task reduce or exaggerate the effects of distracters? Chen and Cave (2006) found that even irrelevant distracters were processed on a task that compared objects by a specific dimension. By manipulating word frequency of an embedded word (a word containing a target letter, e.g., target is A and the embedded word is *CAT*) automatic attention to word processing can be studied in a visual search task. If word recognition occurs, then processing of a target letter within that word should be facilitated because of the preexisting knowledge of how to spell that word. The results of this study would have profound implications for letter search effects by demonstrating that the way in which the distracter is processed is a factor that affects performance much in the same way as distracter feature and target-distracter similarity.

An important question in the study of distracters is what causes distracters to capture attention? Theeuwes (1995) has argued that salient features of distracters in the task (i.e., those which easily stand out from the display) will capture attention in a bottom-up processing fashion. This would be the manner in which targets could be identified in a “pop-out” search task. In a pop-out search, the target is identified rather easily and the effects of distracters are marginal. This is because the target differs by only one feature in a pop-out search (e.g., searching for a *Q* among a distracter array of *O*s of a visual search task).

In traditional visual search studies, different letters are used in a letter string, which serves as distracters. However, in this study the arrangement of distracter letters and target letters to form valid, English words within the letter string created a new type of distracter based upon lexicality and not just letter features. Becker (2008) found evidence of priming of early attentional stages in a visual search task. Unlike Becker, in this study the distracters are not based upon visual features such as orthography, but are based upon lexicality. Findings of changes in performance when target letters are embedded within a word would lend support to arguments in favor of automatic semantic activation (Heil, Rolke, & Pecchinenda, 2004). Furthermore, strong word frequency effects would be indicative of different levels of processing that would affect visual search performance in different ways. A holistic bias of word recognition would impair detection of the target in the visual search task by placing attention on the holistic, word-level processing of the high word frequency. Conversely, low word frequency would assist, or at least not impair, detection of the target by requiring a more analytical and less-holistic processing of the distracter word.

## EXPERIMENT 1

### Hypotheses

In Experiment 1, the first hypothesis that is tested is that high word frequency will cause a detriment to performance because holistic processing interferes with letter-level recognition. Lower word frequency, conversely, should facilitate performance due to the more analytical processing of lower frequency words.

### Method

#### Participants

Forty-three undergraduates (37 female and six male) from the State University of New York at Plattsburgh were recruited for this experiment. The age range of participants was 20 to 43 years of age (*M* = 27.05, *SD* = 6.99). Participants received course credit in exchange for participation and were naďve to the purpose of the experiment.

#### Procedure

All stimuli were presented on Dell 17-in. CRT monitors. Stimuli were presented using E-Prime 2.0 software (Psychology Software Tools, Pittsburgh, PA). Participants performed a visual search task. Prior to each trial a target would be presented on the screen until participants would press the space bar with their nondominant hand to begin the trial. The letter string was presented next and remained on the screen until the participant responded by pressing on a keyboard as to whether the target was present (right arrow key) or absent (the left arrow key) in the letter string.

#### Stimuli

**Figure 1.**
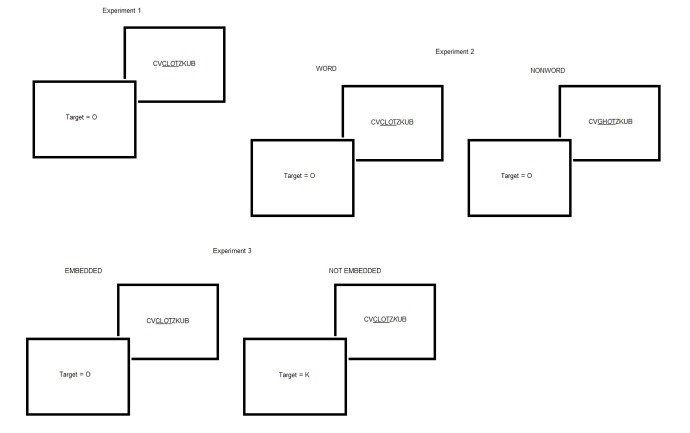
Stimuli display for Experiments 1 through 3. Underline represents word, and italics represents target within the letter string. Neither target nor word were italicized or underlined during Experiments 1 through 3.

Participants completed 10 practice trials before completing 600 trials that were presented in three blocks of 200 trials. One hundred twenty trials were target absent trials, and 480 trials contained a target. Targets were letters and all 26 letters of the English alphabet were used as targets. Using a small set of letters as targets may result in a systematic confusion due to feature similarity (e.g., a target *B* has a feature overlap with *R*). All letters of the English alphabet were used randomly throughout the experiment to prevent any systematic confusion due to feature similarity. Target letter location was randomized throughout the letter strings to reduce predictability. All letter strings (both target present and absent) were exactly 10 letters in length and in Courier New 18 point font (see Figure 1).

Target present trials were divided into three word frequency categories containing 160 trials of nonreplaced words at each word frequency category. Words were extracted from word frequency database of Brysbaert and New (2009). High frequency words occurred between 100 and 20 times per million. Medium frequency occurred less than 20 and more than 10 times per million. Low frequency words were 10 or less occurrences per million. In the target absent condition, no letter string contained a legal, English word. All trials were randomized.

Ktori and Pitchford (2009) observed that English speaking children showed facilitation for identification of letter-positions on the right end of words. Pitchford, Ledgeway, and Masterson (2008) found that in five-letter words there was a benefit to identification of letters in a W pattern. The first, last, and exact middle letters showed facilitation for letter identification in a letter search. For that reason target letters which appeared within a word were placed at random locations throughout the word in order to prevent a position-bias effect.

### Results

All responses less than 300 ms and greater than 2,000 ms were excluded from the analyses. This response latency criterion was used for all subsequent experiments. Data for the three word frequency categories were subjected to a within-subjects (repeated measures) ANOVA.

#### Accuracy

Word frequency had a significant main effect, *F*(2, 84) = 9.50, *p* < .01, η_p_^2^ = .261. Low frequency words had higher accuracy (mean accuracy = 95.2%) than medium frequency (94.2%) and high frequency (94.3%) words.

#### Reaction Time

**Figure 2.**
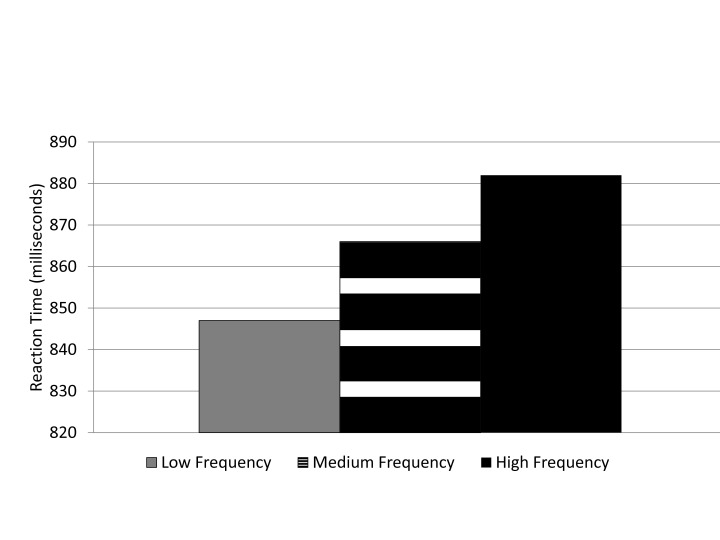
Experiment 1: Mean reaction time (in milliseconds) for word frequency conditions.

Only trials in which an error was not recorded were used for reaction time analysis. Word frequency reaction time had a significant main effect, *F*(2, 84) = 42.28, *p* < .01, η_p_^2^ = .477. Reaction time (low word frequency = 847 ms, medium word frequency = 866 ms, high word frequency = 882 ms) became slower as word frequency increased, which supported Hypothesis 1 (see Figure 2). Normal word frequency effects show a reduction in reaction time as word frequency increases. However, as predicted, the tendency toward holistic processing would be detrimental to target identification in the visual search task, hence slower reaction time for high frequency words compared to low word frequency distracters were found in Experiment 1.

#### Discussion

Experiment 1 demonstrated that reaction time increases when a target letter appears within a high frequency word (confirming Hypothesis 1). These results suggest that holistic bias of high frequency word processing in a visual search task is automatic and interferes with letter-level feature search. This reverse word frequency effect suggests that automatic processing of distracters occurred even when the distracter was not relevant to the task.

Carr, Posner, Pollatsek, and Snyder (1979) reported that orthography facilitated all levels of word processing, but the effect of familiarity was confined to lexical performance. High frequency words are encountered often enough to be seen as familiar. Hence, high frequency word distracters and the accompanying holistic bias serve to confine processing at a lexical level and not a letter level where performance would be affected by orthography.

### EXPERIMENT 2

#### Introduction

The use of words as distracters in a letter search task indicates that lexicality (e.g., word frequency effects) represents a distracter dimension that is processed automatically and affects performance. If words as distracters affect performance, how would that performance differ if no lexical distracters are present? Experiment 2 was designed to test if the effects of lexical distracters (although affected by word frequency/familiarity) have a performance cost compared to a condition where no lexical distracters are present (effectively a nonword condition) that would serve as a baseline of performance.

Automatic processing of words may have a beneficial effect. A target embedded in a word may be processed faster than a target not embedded in a word. The bias for automatic word processing may serve to capture attention. Comparison of letter strings containing a word to letter strings not containing a word would allow a closer examination of attention capture by word distracters.

#### Hypothesis

Hypothesis 2: In Experiment 2, not only will Hypothesis 1 be replicated, but there will be a distinct advantage for letter strings containing word distracters over letter strings not containing words. Although high frequency words will be related to poorer visual search performance, compared to medium and low frequency words, lexicality (although task irrelevant) will facilitate performance over nonwords.

#### Method

#### Participants

A new group of participants were recruited for Experiment 2. Participants were 44 undergraduates (five male and 39 female) from the State University of New York at Plattsburgh. The age range of participants was 20 to 48 years of age (*M* = 27.77, *SD* = 7.89). Participants received course credit in exchange for participation.

#### Stimulus

All stimuli were identical to Experiment 1 with the exception that in Experiment 2 there was now a category for nonwords. Nonword generation is described below.

#### Procedure

Participants completed 10 practice trials followed by 600 trials that were presented in three blocks of 200 trials. One hundred twenty trials were target absent trials and 480 trials contained a target. Target present trials were divided into two categories: One category had the target embedded within a word, and one category had the target appear in a letter string that did not contain a valid word, hereafter referred to as the nonword category. There were 72 nonword trials in Experiment 2. This preserved the same number of trials from Experiment 1 and reduced the risk of fatigue. Nonwords were created by replacing a single letter from a valid word. The letter that was replaced was randomized. The word category was identical to the word category from Experiment 1, except that 136 words were used per word frequency condition. All trials were randomized.

### Results

#### Accuracy

There was a significant effect of word/nonword, *t*(43) = 3.73, *p* < .01. Nonword trials had a mean accuracy of 88.9%, while word trials had a mean accuracy of 90.1%. The main effect of word frequency was marginally significant, *F*(2, 86) = 2.78, *p* = .068, η_p_^2^ = .139. Medium frequency words had lower mean accuracy (90.2%) than low frequency (89.6%) and high frequency (90.4%) words.

#### Reaction Time

**Figure 3.**
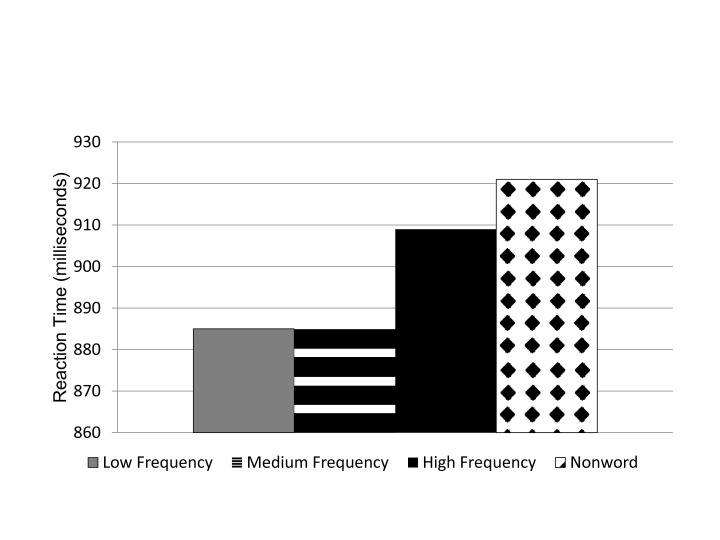
Experiment 2: Mean reaction time (in milliseconds) for nonword and word frequency conditions.

There was a significant main effect for word frequency, *F*(2, 86) = 6.62,p < .05, η_p_^2^ = .093. The results of Experiment 1 were replicated because Experiment 2 also demonstrated slower mean reaction time for high word frequency (909 ms) than for medium (885 ms) and low (885 ms) word frequency (see Figure 3). The comparisons of words to nonwords had a significant main effect, *F*(1, 43) = 20.44, *p* < .05, η_p_^2^ = .349. Trials with a target embedded in a valid word had a mean reaction time of 893 ms, while trials with a target in a letter string that contained no valid words (nonword) had a slower mean reaction time (921 ms). This supported Hypothesis 2 in that words would facilitate performance by allowing easier access to relevant letter-level information.

### Discussion

The results of Experiment 2 show that when a target is present within a word distracter in a letter string there is a benefit to performance. This advantage over nonword distracters supports the findings of Reicher (1969). High frequency words had longer reaction times than medium and low frequency words. Most crucial to Experiment 2 was that nonwords had longer reaction times than high frequency words, which supported Hypothesis 2. Although a holistic bias does affect performance, the use of words as distracters captures attention and facilitates letter search performance.

## EXPERIMENT 3

### Introduction

In Experiment 1 target letters were presented within a word, and in Experiment 2 target letters were presented within either a word or a nonword. In both experiments words served to capture attention and facilitate letter search performance (albeit to different levels of facilitation depending on word frequency). Would the same effect for word distracters occur in trials in which the target letter did not appear within a distracter word? In Experiment 3 words were now used as distracters in two conditions. In one condition the target was presented within a word (as in Experiments 1 and 2). The second condition had a legal word within a letter string, but the target letter that was present was not within the word distracter (a nonembedded word distracter; see Figure 1). This allows for a comparison of word distracters capturing the focus of attention either towards or away from a target letter.

Experiments 1 and 2 showed impaired letter-level processing of high frequency words compared to lower frequency words due to holistic bias. Indeed, Wheeler (1970) showed a benefit for letter search when the letter string formed a word. An explanation for the results of Experiments 1and 2 is that the target being embedded within a distracter word could also serve to localize/capture attention toward the target letter. If the distracter word served to capture attention which facilitated detection of the target letter, could the capture of attention by word distracters still produce an effect on performance when the target is not within the distracter word? Experiment 3 will serve to examine if the word frequency of lexical distracters is a distracter dimension which is not entirely dependent upon the proximity between targets and distracters.

### Hypotheses

Hypothesis 3: In letter strings containing a distracter word, targets that are embedded within the distracter word will have shorter reaction times than targets that are not embedded within the distracter word. This will arise because word bias will draw attention toward the distracter word which would facilitate detection for a target that is embedded within the distracter word.

Hypothesis 4: Targets within a high frequency word will have longer reaction times than targets in lower frequency words. This is a result of the holistic bias of word recognition as stated in Hypotheses 1 and 3.

### Method

Participants for Experiment 3 were 23 undergraduates (two male and 21 female) from the State University of New York at Plattsburgh. The age range of participants was 20 to 45 years of age (*M* = 25.91, *SD* = 8.48).Participants received course credit in exchange for participation.

#### Stimuli

Experiment 3 was identical to Experiment 2 with the exception that now the target letter could be embedded within a word or the target could be presented within a letter string, but not be embedded within a word (see Figure 1). This created an embedded condition where a target (such as *U*) could be embedded within a word such as *PUT*. This would produce a trial where the letter string might look like *XRPUTMOCD*. Conversely, in the nonembedded condition, the target does not appear inside a word, but the word is present in the letter string and serves as a distracter. For example, if the target is C and the letter string is *RPUTMOCD*, then *PUT* serves as a word distracter. Target and distracter locations were randomized to reduce predictability by the participants.

There were 600 trials in Experiment 3. One hundred-twenty trials did not contain a target. In the remaining 480 trials, 72 trials served as nonword control trials in which a target letter was present, but no legal, English word was present in the letter string. Two hundred-four trials had a target letter and word distracter, but the target letter was not embedded within the word distracter. Word distracters were divided into the three word frequency groups as in Experiment 1 which resulted in 68 trials of targets not embedded within a word per word frequency category. The remaining 204 trials had target letters embedded within a word divided by the previously mentioned word frequency categories.

#### Procedure

The procedure was the same as Experiment 2, except for the stimuli changes mentioned above.

### Results

#### Accuracy

Data was analyzed in a 2 (Embedding: embedded targets vs. nonembedded targets) × 3 (Word Frequency: low, medium, high) ANOVA. There was a significant main effect for embedding, *F*(1, 22) = 8.65, *p* < .05, η_p_^2^ = .345. The main effect for word frequency was significant, *F*(2, 44) = 4.79, *p* < .05, η_p_^2^ = .257. There was a significant interaction between Embedding and Word Frequency, *F*(2, 44) = 6.32, *p* < .05, η_p_^2^ = .207. This interaction was the result of higher accuracy for targets embedded in low frequency words over nonembedded low frequency words, while at higher word frequencies the effect of embedded targets was smaller.

#### Reaction Time

**Figure 4.**
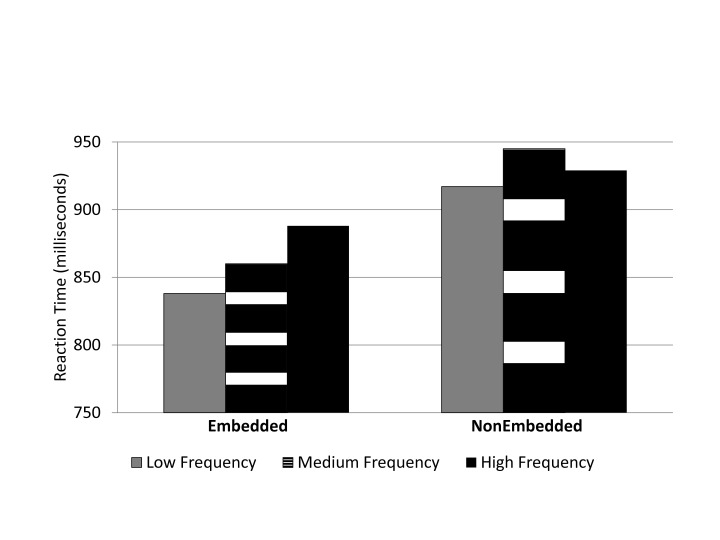
Experiment 3: Mean reaction time (in milliseconds) for letter strings with targets embedded within a word and targets not embedded within a word

Data was analyzed in a 2 (Embedding: embedded targets vs. nonembedded targets) × 3 (Word Frequency: low, medium, high) ANOVA. There was a significant main effect for embedding, *F*(1, 22) = 62.06, *p* < .05, η_p_^2^ = .741. Letter strings with target letters embedded within words had shorter mean reaction times (841 ms) than letter strings with target letters that were not embedded in distracter words (893 ms). The main effect for word frequency was significant, *F*(2, 44) = 10.77, *p* < .05,η_p_^2^ = .332. There was a significant interaction between Embedding and Word Frequency, *F*(2, 44) = 4.84, *p* < .05, η_p_^2^ = .177. This occurred because when targets in a letter string were embedded within words, the reaction times followed a typical word frequency effect (see Figure 4). Follow up *t*-tests of the embedded condition revealed a significant difference between low and medium frequency words, *t*(22) = 2.02, *p* < .05; medium and high frequency, *t*(22) = 2.50, *p* < .05; and low and high frequency words, *t*(22) = 7.13, *p* < .05. In the nonembedded condition, the only significant different among word frequency was between low and medium frequency word, *t*(22) = 3.46, *p* < .05.

A *t*-test comparing word and nonword conditions showed a significant reaction time advantage for nonwords (mean RT = 874 ms) over words (897 ms), *t*(22) = 2.74, *p* < .05. This reversal of the advantage of words over nonwords in Experiment 2 is a result of the embedded and nonembedded conditions. The nonembedded condition (which was not realized in Experiment 2) showed a significant reaction time disadvantage for targets not embedded in words (931 ms) compared to nonwords (874 ms), *t*(22) = 5.22, *p* < .05. However, the previous advantage of targets embedded in words (863 ms) over nonwords (874 ms) was found, but only approached marginal significance (*p* = .127).

### Discussion

Hypothesis 3 was confirmed by Experiment 3’s results showing an advantage for targets embedded within a word over targets not embedded in a word. Word distracters in the nonembedded condition did not show the reverse word frequency effect that embedded word distracters showed in Experiments 1, 2, and 3. The results of Experiment 3 also confirmed Hypothesis 4 which showed that targets embedded within high frequency words had longer reaction times than lower frequency words. This effect was not as distinct for words that did not have embedded targets, which suggests that proximity to the target may slightly affect automatic lexical processing. Embedding a target within a word facilitates access to letter-level processing, but is still negatively affected by holistic bias.

## GENERAL DISCUSSION

The results of three experiments showed that when performing a letter search task, embedding the target within a word influenced reaction times. This benefit of word distracters is consistent with Heil et al.’s (2004) evidence of automatic semantic activation in a letter search task and contrasts with Stolz and Besner’s (1996) and Friedrich et al.’s (1991) suggestion that semantic activation is nonautomatic. The benefit of valid words over random, nonlexical letter strings is consistent with the results of the word superiority effect of Wheeler (1970). However, a reverse word frequency effect was discovered for targets embedded within distracter words. This slowing as word frequency increased was predicted to occur for high frequency words which are more likely to be processed holistically. These results show holistic lexical proces-sing of distracters in a nonlexical task and supported Hypothesis 1. In Experiment 2, Hypothesis 2 was supported by evidence showing that although targets embedded in high frequency words had the poorest reaction times compared to low and medium word frequency distracters, a lexicality advantage in visual search for targets embedded within words over targets embedded in nonwords. Experiment 3 showed how word distracters can capture attention toward or away from a target depending upon whether the target is embedded within the distracter word.

Studies of distracter effects have shown a greater detriment to performance when distracters shared a common dimension or feature with the target (Chen & Cave, 2006; Flowers & Lohr, 1985). The findings of this study are unique in that it was a task-irrelevant dimension of lexicality and not feature similarity that affected performance. Lexicality improved performance, but showed a reduced benefit at high word frequency, which was due to holistic bias. This showed a strong distracter effect based upon how the distracter is processed (holistic vs. letter-level) rather than the distracter feature.

The results of this study give a novel contribution to distracter research by showing that a lexical distracter can affect letter search performance. Previous studies, like by Neo and Chua (2006), Küper and Heil (2009), Chen and Cave (2006), and by Flowers and Lohr (1985), showed location and timing were major distracter factors. Now it can be observed that lexical effects (such as word frequency) in a visual search task can facilitate performance over non lexical information (Experiment 2), but still impair performance by holistic processing and embedded targets (Experiments 1 and 3).

A stochastic race between letter-level and holistic, word-level processing provides a model for how the distracters of the visual search task can lead to pop-out effects. Allen et al. (1995) found evidence of a benefit for holistic-word processing over letter-level processing. This would suggest that the results are from word-level, holistic processing attaining a speed advantage and impairing letter-level processing akin to different levels of processing much like the results of Neo and Chua (2006) and of Bacon and Egeth (1994).

A question for the area of visual search raised by this study is: What influence do the task demands have in suppressing the effects of distracters? The evidence suggests that task demands do not have a strong impact on preventing distracter influences. Flowers and Smith (1998) showed that when distracters are switched, there is a detriment to reaction time performance. This occurs even when participants are not aware of the switch, which suggests that distracter effects can occur implicitly (a relevant point for a visual search task with embedded words). Although task demands in a visual search task require selectivity of attention, distracters were able to impair search performance on a task-irrelevant dimension. This finding supports previous studies that demonstrate that semantic activation is an automatic process (Heil et al., 2004). Furthermore, the findings of word frequency effects in all three experiments indicate a more thorough level of lexical distracter processing (a depth of processing effect akin to Smith, Theodor, & Franklin, 1983) than previous studies have shown. This lexical dimension was not only irrelevant to the task, but was processing holistic information which was different from the letter-level, orthographic similarity between the target and distracters.

To answer the question asked earlier, if the target and distracters were similar, but the distracters formed a word (with the target embedded within the word), would the irrelevant dimension of lexicality affect performance? Lexicality improved search performance by allowing access to letter-level information through automatic word re-cognition. However, the greater the ease of lexical access, such as with high frequency words, the greater the distraction on the letter-level/orthographic visual search. Neo and Chua’s (2006) third experiment showed that distracters could be detrimental to performance even if the location of the target was known (this was valid when the onset of distracters was under 200 ms). Their results showed that task demands did not prevent distracter effects. In conditions where the target location was already known and onset was greater than 200 ms, top-down processing could prevent bottom-up distracter influence from being detrimental to performance. The results of this study show how distracters can cause a top-down detriment (holistic bias) to performance despite being irrelevant to task demands. Both Neo and Chua’s study and Experiments 1-3 show task demands did not eliminate distracter influence, but unlike Neo and Chua this study showed that it was the opposite influence in that a top-down, not a bottom-up process caused the detriment to performance.

Relevant to the question of whether automatic word form processing would create a scenario of anti-axiomatic “not being able to see the trees because of the forest” the results of Experiments 2 and 3 produce evidence to suggest: no, not really. Experiment 2 showed again a detriment for targets embedded within a high frequency word which supported Hypothesis 1. Hypothesis 3, however, answers the “trees or forest” question by showing (as suggested by the results of Experiments 2 and 3) that target letters embedded within a word had faster reaction times than letters embedded within a nonword. In this case automatic word processing served to capture attention *toward* the type of processing necessary for target detection. Being able to automatically process a word form allowed for easier access of letter-level information.

Many studies of distracter influence have examined the effects of distracters from the perspective of lower-level processing affecting higher-level processing (Bacon & Egeth, 1994). This study is unique in that the distracters influenced an automatic, high-level lexical process, which impaired a letter-level visual search task. The novel implication is that although targets and distracters shared a similar dimension (orthography) it was the task-irrelevant and stimulus-irrelevant word frequency that led to impairment in the overall visual search performance, despite task demands.
